# One Health Surveillance: A Matrix to Evaluate Multisectoral Collaboration

**DOI:** 10.3389/fvets.2019.00109

**Published:** 2019-04-24

**Authors:** Marion Bordier, Camille Delavenne, Dung Thuy Thi Nguyen, Flavie Luce Goutard, Pascal Hendrikx

**Affiliations:** ^1^Centre de Coopération Internationale en Recherche Agronomique Pour le Développement, UMR ASTRE, Hanoi, Vietnam; ^2^ASTRE, Univ Montpellier, CIRAD, INRA, Montpellier, France; ^3^National Institute of Veterinary Research, Hanoi, Vietnam; ^4^CIRAD, UMR ASTRE, Bangkok, Thailand; ^5^Faculty of Veterinary Medicine, Kasetsart University, Bangkok, Thailand; ^6^French Agency for Food, Environmental and Occupational Health Safety (ANSES), UCAS, Lyon, France

**Keywords:** collaboration, evaluation, multisectoral, one health, surveillance

## Abstract

The international community and governmental organizations are actively calling for the implementation of One Health (OH) surveillance systems to target health hazards that involve humans, animals, and their environment. In our view, the main characteristic of a OH surveillance system is the collaboration across institutions and disciplines operating within the different sectors to plan, coordinate, and implement the surveillance process. However, the multisectoral organizational models and possible collaborative modalities implemented throughout the surveillance process are multi-fold and depend on the objective and context of the surveillance. The purpose of this study is to define a matrix to evaluate the quality and appropriateness of multisectoral collaboration through an in-depth analysis of its organization, implementation, and functions. We developed a first list of evaluation attributes based on (i) the characteristics of the organization, implementation, and functionality of multisectoral surveillance systems; and (ii) the existing attributes for the evaluation of health surveillance systems and OH initiatives. These attributes were submitted to two rounds of expert-opinion elicitation for review and validation. The final list of attributes consisted of 23 organizational attributes and 9 functional attributes, to which 3 organizational indexes were added measuring the overall organization of collaboration. We then defined 75 criteria to evaluate the level of satisfaction for the attributes and indexes. The criteria were scored following a four-tiered scoring grid. Graphical representations allowed for an easy overview of the evaluation results for both attributes and indexes. This evaluation matrix is the first to allow an in-depth analysis of collaboration in a multisectoral surveillance system and is the preliminary step toward the creation of a fully standalone tool for the evaluation of collaboration. After its practical application and adaptability to different contexts are field-tested, this tool could be very useful in identifying the strengths and weaknesses of collaboration occurring in a multisectoral surveillance system.

## Introduction

After dividing and categorizing knowledge into disciplines for years, the growing concern around complex health hazards urges us to reconsider the need for systemic and holistic approaches to better face these new challenges (1). This is in line with the One Health (OH) concept that promotes the de compartmentalization of human, animal, and ecosystem health for a more efficient and sustainable governance of complex health issues (2, 3). To this end, international, national, and local efforts are increasing to support the establishment and implementation of OH surveillance systems to more effectively manage health hazards at the human—animal—environment interface (4).

Although there is currently no consensual definition of OH surveillance, collaborative efforts across sectors and disciplines are at the heart of definitions found in the literature (5–8). However, there is a broad spectrum of possible organizational models for the governance of collaboration, and its operationalization varies in terms of areas of implementation throughout the surveillance process, and in terms of intensity. The collaborative setting is mainly driven by the surveillance context and objective, and is built according to stakeholders' constraints and expectations. Only a proper evaluation of collaboration, supported by a rigorous and adequate methodological framework, could assess whether collaborative efforts are appropriate and functional, and whether they improve the value of surveillance (9).

Despite the fact that collaboration is a key factor in the implementation of OH surveillance, no evaluation method currently focuses on (i) the quality of multisectoral and interdisciplinary collaboration, or (ii) the measurement of impacts and benefits resulting from collaborative surveillance as compared to a juxtaposition of isolated sectoral surveillance components. The current evaluation tools for surveillance systems do not consider collaboration in depth (10). Furthermore, evaluation frameworks focusing on OH initiatives, including OH surveillance, evaluate the structural balance of the initiative compared to an ideal OH approach, as well as its outcomes, rather than the quality of collaboration itself (11).

The quality of the information produced by a surveillance system depends on the quality of its organization (12). Hence, we argue that the evaluation of the organization, implementation, and functionality of collaboration is crucial to attest to the surveillance system's capacity to produce relevant information.

The aim of this study is to develop a matrix with which to evaluate collaboration in multisectoral surveillance systems, where collaborative efforts are deployed across institutions operating in several sectors, but equally across diverse disciplines, decision-making scales, and professions, to address complex health hazards. Based on a literature review and expert-opinion elicitation, we first identified a list of attributes and indexes that characterize the organization, implementation, and functionality of collaboration taking place in any multisectoral surveillance system. Then, we developed a scoring grid to obtain evaluation results for these attributes and indexes.

## Materials and Methods

The evaluation matrix for collaboration taking place in a multisectoral surveillance system was developed in two main phases: the identification of evaluation attributes and indexes, followed by the development of the scoring method to obtain semiquantitative evaluation results.

**Figure 1:**
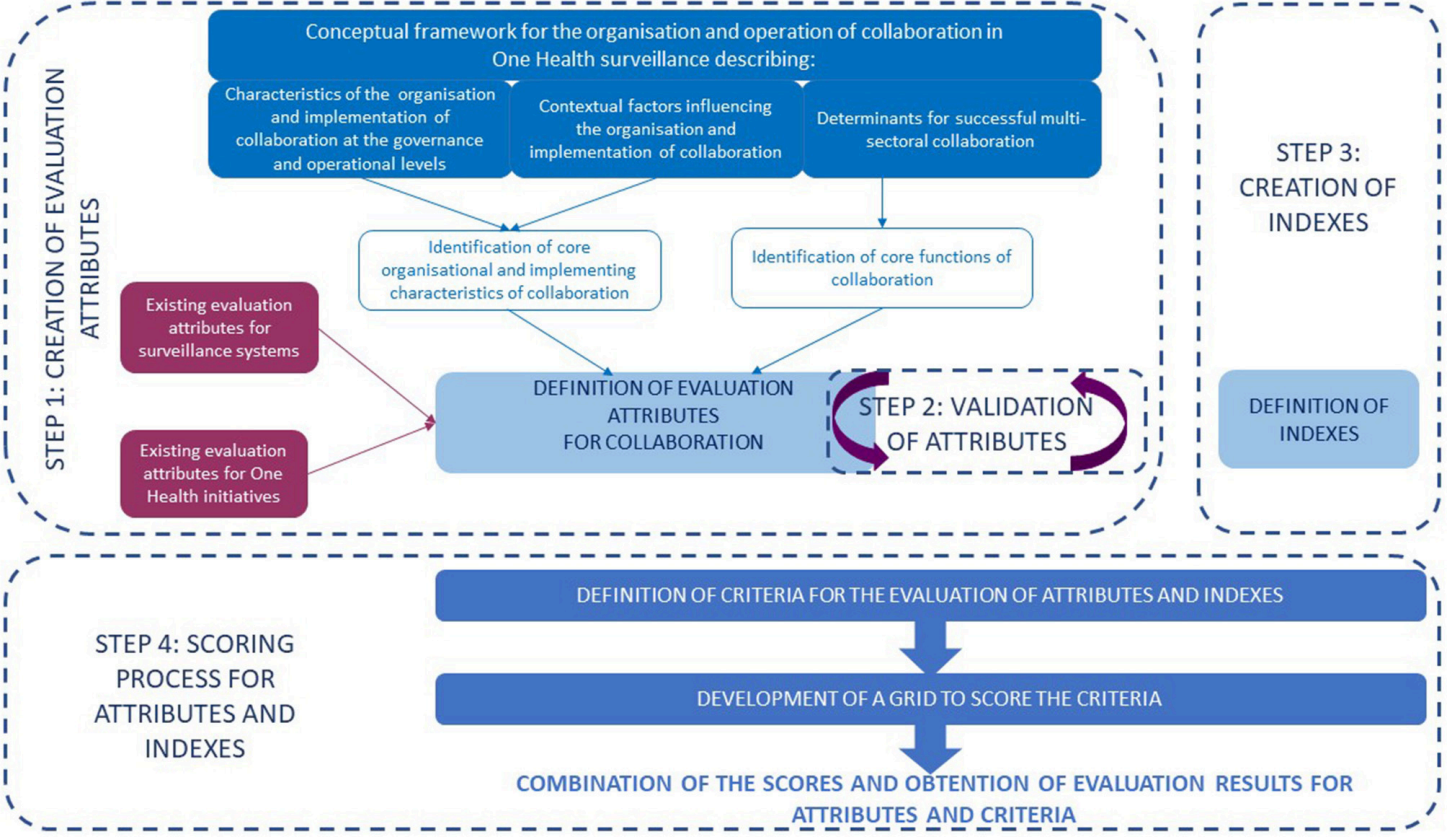
The methodological framework for the development of the evaluation matrix.

Figure 1 provides an overview of the methodological framework, which is detailed in the following four steps.

### Step 1: Identification of Specific Evaluation Attributes for Collaboration in a Multisectoral Surveillance System

The identification of evaluation attributes for collaboration taking place in a multisectoral surveillance system was based on three sources of information: (i) a conceptual framework to characterize the organization and implementation of OH surveillance systems, (ii) existing OH evaluation frameworks, and (iii) existing surveillance system evaluation tools.

**Figure 2:**
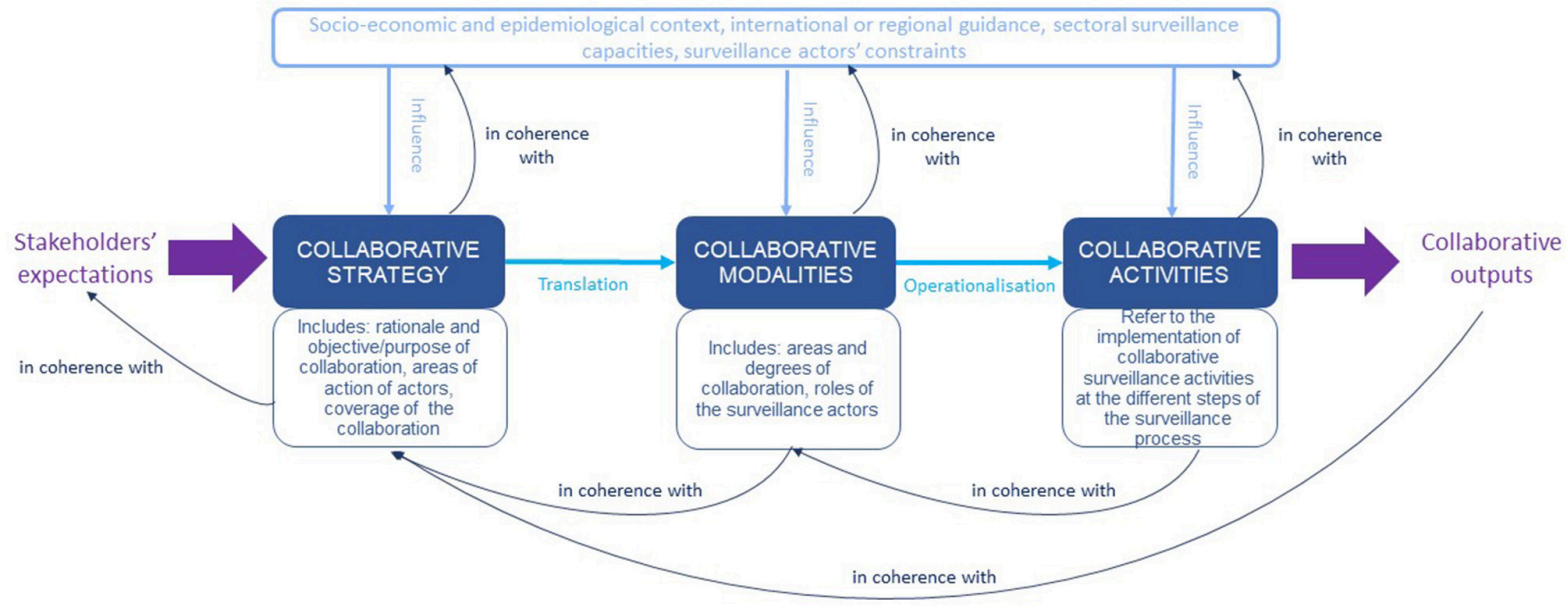
A conceptual framework for the organization and functioning of collaboration in a one health surveillance system.

Our starting point was the conceptual framework for the characterization of collaboration in a OH surveillance system, proposed by Bordier et al. (13). Figure 2 is an adapted representation of this framework, which identifies three levels of collaboration: the policy level where the collaborative strategy is enunciated; the institutional level where relevant collaborative modalities are defined to achieve the desired goals of the strategy; and the operational level where surveillance activities are implemented to ensure the routine operation of collaborative modalities. The three levels of collaboration must be clearly formalized and endorsed by stakeholders and be relevant with regard to each other. Collaboration for surveillance is generated by stakeholders' expectations under the influence of a broad range of contextual factors. Collaborative activities throughout the surveillance process lead to the production of outputs (up-to-date information, multistakeholder network, etc.). That must meet the collaboration's objective and purpose.

This framework sets down the core characteristics for the organization and implementation of collaboration at governance and implementation levels, as well as the contextual factors that influence them, and identifies core functions for a successful multisectoral surveillance system.

Information provided within this framework was then compared with evaluation attributes for surveillance systems used in EvaTool (14) and Oasis (12) and with evaluation frameworks for OH, namely, the Network for Evaluation of One Health (NEOH) framework, to conceptualize and conduct evaluations of integrated approaches to health (11); and the One Health Assessment for Planning & Performance (OH-APP tool)^1^, to assess the organizational capacity and performance of a multisectoral coordination mechanism.

A back-and-forth process between the different information sources was carried out to identify specific attributes that must be considered in order to accurately evaluate collaboration taking place within a multisectoral surveillance system.

### Step 2: Validation of the Attributes for the Evaluation of Collaboration Within a Multisectoral Surveillance System

The list of attributes for the evaluation of collaboration within a multisectoral surveillance system was validated by a panel of international experts in a two-round process.

For the first round, expert opinions were elicited using an electronic questionnaire developed with the Surveymonkey™ tool. Selected experts were the authors of articles related to OH surveillance or had been involved in research consortiums working either on integrated surveillance evaluation (Risksur project) or on OH evaluation (NEOH). A total of 256 experts were contacted by email to take part in the study by answering the online questionnaire. Primary recipients were asked to freely forward the questionnaire to anyone in their network with an interest in OH surveillance. The questionnaire was also advertised on LinkedIn. The questionnaire included three main sections: (i) personal information, (ii) characterization of OH surveillance, and (iii) evaluation of collaboration. Only the first and third sections of the questionnaire were considered for this study. In the first section, participants were asked to provide information on their working institution, academic background, and expertise and experience in OH surveillance. In the third section, they were asked if they considered all proposed evaluation attributes for collaboration to be relevant and if they could identify any missing attributes. Participants had to answer yes/no questions and justify their choice in an open box. The questionnaire was tested through two pilot interviews to assess the questions' clarity and was freely accessible online from March 9 to 30th, 2018. Experts' answers were then uploaded into an Excel spreadsheet, in which each row corresponded to a participant identified by his/her name and IP address, and each column to their answer for each question. A descriptive study was conducted on experts' backgrounds and opinions on the list of attributes (relevant or missing). Open comments and justifications regarding attributes were analyzed and categorized.

Based on experts' answers and comments, the initial list of evaluation attributes was refined. A second round of expert-opinion elicitation was then organized with a restricted number of experts who had expressed detailed comments during the first elicitation round. The objective was to discuss, through a video conference, certain specific points that were highly commented during the first round and for which we wanted feedback on the way we proposed to address them, namely: (i) information sharing and system knowledge, (ii) leadership, and (iii) functional attributes for collaboration. The meeting took around 90 min. The discussion was recorded for further analysis and the resulting conclusions were used to validate or refine the list of attributes and their definitions.

### Step 3: Creation of Indexes for the Organization of Collaboration

Along with the identification of evaluation attributes, we developed indexes that provide an overview of the general organization of collaboration. In contrast to attributes, which relate to a specific organizational characteristic of collaboration, indexes aim at reflecting the collaboration's organization at a macro level.

Our reasoning was based on the process approach to quality management in companies, including testing laboratories^2^. In this model, the company is modeled as a series of interlinked processes that transform clients' needs and expectations into a deliverable, which can be a product or a service, through the implementation of activities. Three processes are defined, namely:

- The operation process, which represents activities that generate the product or the service;
- The support process, which represents activities that ensure the smooth operation of the company, such as financial and human resources, training, communication, etc.;
- The management process, which represents activities implemented to ensure the company meets its goals.

A surveillance system can be conceptualized following the same approach. It can be represented as an organization whose goal is to provide surveillance results in accordance with stakeholder demands and expectations, through the implementation of surveillance activities (operation process). The model can be restricted to collaboration taking place in a multisectoral surveillance system, where inputs are stakeholders' expectations regarding collaboration and outputs are the results obtained thanks to the collaborative effort.

We created indexes to measure the level of satisfaction of all activities contributing to each process.

### Step 4: Scoring Process for Attributes and Indexes

The scoring methodology was largely inspired by that used in the Oasis tool, in which assessment criteria are scored to semiquantitatively measure evaluation attributes and critical control points (12).

Once specific attributes and indexes for collaboration were defined, we singled out the necessary criteria to support their evaluation. To this end, we identified specific elements included in the definition of attributes and indexes and formulated them as criteria for the further evaluation of the latter. A semiquantitative four-tiered scoring scale was established to score each evaluation criterion depending on the level of fulfillment achieved by the collaborative situation under evaluation. Four grades were defined: grade 3 indicates that collaboration complies fully with the criterion, while grade 0 indicates a total absence of compliance; grades 2 and 1 are intermediate grades depending on the level of compliance. In some cases, the value “Non-relevant” can be used if the criterion is not relevant to the multisectoral surveillance system under evaluation. For each grade, a scoring guide was developed to describe the situation in which they should be awarded.

Grades of attributes and indexes are obtained by combining grades awarded to the criteria supporting their definition. “Non-relevant” values, if there are any, do not impact the final grade.

## Results

### The Initial List of Evaluation Attributes

Based on the analysis of the three information sources, we first identified 38 attributes relevant to the evaluation of collaboration in a multisectoral surveillance system. These attributes were categorized into five groups: governance (9), operation (8), effectiveness (10), function (7), and value (4). Table 1 presents the detailed list of attributes.

**Table 1:** List of attributes submitted to the first round of expert-opinion elicitation.

**Governance attributes**	**Operational attributes**	**Function attributes**	**Effectiveness attributes**	**Value attributes**
Formalization at the policy level Formalization at the institutional level Relevance of the collaborative objective Relevance of the collaborative modalities Mechanisms Resources Performance and evaluation Training Information	Appropriate collaborative activities and availability of related resources for planning Appropriate collaborative activities and availability of related resources data collection Appropriate collaborative activities and availability of related resources for laboratory testing Appropriate collaborative activities and availability of related resources for data management and storage Appropriate collaborative activities and availability of related resources for data exchange Appropriate collaborative activities and availability of related resources for data analysis and interpretation Appropriate collaborative activities and availability of related resources for communication Appropriate collaborative activities and availability of related resources dissemination	Stability and sustainability Acceptability and engagement Simplicity Adaptability and flexibility Portability Interoperability Data completeness and correctness	Coverage Exhaustiveness Representativeness False alarm rate Precision Timeliness Sensitivity Positive predictive value Negative predictive value Repeatability	Cost Technical impact Benefit Economic acceptability

Each group of attributes focused on the evaluation of a different aspect of collaboration: governance and operation attributes on its organization; effectiveness attributes on its impact on surveillance performance in each sector covered by the multisectoral surveillance system; functional attributes on the qualities of core collaboration functions required for an effective multisectoral surveillance system; and value attributes on its impacts, benefits, and cost.

### Results From Expert-Opinion Elicitation

The initial list was submitted to expert-opinion elicitation. In total, 84 experts accessed the questionnaire. Only the 39 respondents who fully completed the questionnaire and filled in the section related to evaluation attributes were considered for the study. Most of them were epidemiologists (74%) and/or veterinarians (72%), working mainly in research institutes, universities, or expertise agencies (54%), intergovernmental agencies (21%), or national authorities (18%). Remaining respondents worked in the private sector (13%) or in non-governmental organizations (1%). Respondents' main fields of expertise included epidemiology (95%), veterinary public health (77%), public health (67%), and food safety (61%). Most participants had substantial experience in health surveillance (56%) and in the OH concept (66%). Most of them (85%) demonstrated at least 1 year of experience in OH surveillance. All proposed attributes were considered relevant by 67% of the respondents, and 43% of them answered that no attributes were missing. We did not receive comments on the proposed list from 24% of the respondents. However, we received 61 comments from experts that were related to missing attributes (49% of comments), the need to prioritize attributes (16%), the need to clarify certain attributes (13%), non-relevant attributes (13%), and the need to distinguish attributes related to collaboration from those related to surveillance (8%).

Among the 19 experts contacted to take part in the second round of opinion elicitation, 9 were able to participate in a video conference. Participants were mostly epidemiologists (89%) and veterinarians (89%), mainly working in research institutes, universities or expertise agencies (67%), national authorities (22%), or intergovernmental agencies (22%). One participant worked in the private sector. They mainly had more than 1 year of experience in surveillance (89%), and all had at least 1 year of experience in the field of OH. Globally, 89% of the respondents have at least 1 year of experience in surveillance and OH.

### Main Amendments Brought to the Attributes

Based on expert inputs, we made major amendments to the initial list of evaluation attributes, of which the main ones are described below.

Regarding organizational attributes at the governance level, “Information” was renamed “Information and communication” to address how the information concerning and produced by the multisectoral surveillance system is stored and communicated to surveillance actors and end-users. Additionally, we split the “Mechanisms” attribute into three attributes to distinguish the different governance mechanisms, namely, steering, coordination, and technical and scientific support. At the operational level, where attributes were defined to evaluate the operationalization of collaborative modalities, the attribute related to data exchange was split into two attributes to distinguish the sharing of raw data from the sharing of surveillance results. Indeed, these two modalities are possible and independent from each other, and must therefore be distinguished in the evaluation process.

Deep changes were also brought to the functional attributes to avoid any ambiguity regarding the fact that they were specific to collaboration only and to focus on core characteristics of functional collaboration. Four attributes were discarded, namely, “Simplicity,” “Portability,” “Interoperability,” and “Data completeness and correctness,” as they were not considered to be core collaborative features. Conversely, six attributes were added to the list, to address key functions of collaboration: “Relevance,” “Operationality,” “Resources,” “Inclusiveness,” “Shared leadership,” and “System knowledge.” Finally, three attributes were renamed and their definition slightly refined: “Sustainability” was replaced by “Stability,” “Acceptability and engagement” by “Acceptability,” and “Adaptability and flexibility” by “Adaptability.” These attributes are highly interdependent and contribute to the effectiveness of the multisectoral surveillance system.

Finally, attributes related to effectiveness were not retained as they did not specifically evaluate collaboration but rather the entire surveillance system, including sectoral surveillance. Additionally, value attributes were discarded from the study as their evaluation required additional data and information, which were not available at this stage of the evaluation matrix's development.

The final list of attributes, including 23 organizational attributes (13 for governance and 10 for operation) and 9 functional attributes, is presented in Table 2. Their detailed definition is available in Supplementary Tables 1, 2.

**Table 2:** Final list of organizational and functional attributes.

**Organizational attributes**		**Functional attributes**
**Governance level**	**Operational level**	
G.1 Formalization and endorsement of the collaborative surveillance strategy G.2 Relevance of collaborative objective(s) and purpose G.3 Formalization of collaborative modalities G.4 Relevance of collaborative modalities G.5 Coverage G.6 Governance of resources for collaboration G.7 Mechanism(s) for steering collaboration G.8 Mechanism(s) for coordinating collaboration G.9 Mechanism(s) for technically and scientifically supporting collaboration G.10 Training G.11 Information and communication G.12 Performance and evaluation G.13 Engagement	O.1 Collaboration for surveillance design O.2 Collaboration for sampling O.3 Collaboration for laboratory testing O.4 Collaboration for data sharing O.5 Collaboration for sharing surveillance results O.6 Collaboration for data management and storage O.7 Collaboration for data analysis and interpretation O.8 Collaboration for communication to surveillance actors O.9 Collaboration for external communication O.10 Collaboration for dissemination to beneficiaries	Stability Relevance Operationality Acceptability Resources Adaptability Inclusiveness Shared leadership System knowledge

### Collaborative Organization Indexes

Along with the development of collaboration attributes and based on the process approach for quality management, we defined three indexes that support the macro-evaluation of the organization of collaboration.

- The operation index, which refers to collaborative activities throughout the surveillance process (from surveillance planning to results dissemination) that generate the relevant collaborative outputs to meet the collaborative objective.
- The support index, which refers to elements intended to ensure the smooth operation of collaboration: resources allocation, training, information and communication, technical, and scientific support.
- The management index, which refers to elements that contribute to the management of collaboration: existence and formalization of a collaborative strategy; governance mechanisms for steering and coordination; and performance monitoring and evaluation.

### The Evaluation Criteria for Attributes and Indexes

Based on the identification of elements required to characterize the 32 attributes and the 3 indexes, we developed 75 criteria to evaluate them. The same criterion can be used to evaluate several functional attributes. On the contrary, each organizational attribute and index is evaluated with a set of specific criteria without any overlap. These criteria were refined following changes in attributes brought by the expert-opinion elicitation and to capture certain experts' comments.

Supplementary Tables 1–3 provides the list of criteria that support the evaluation of each attribute and index (Supplementary Materials).

Certain notions were particularly underlined during the expert-opinion elicitation and we paid specific attention to their evaluation.

First, we introduced the notion of “feedback loop” for which we developed a specific criterion. This notion refers to the fact that the outputs of the surveillance system and lessons learnt (previous evaluation results, feedback from operational actors, etc.) are routed back to the governance mechanisms where they are used as inputs to inform decisions and to adapt to changes. Criteria related to the existence of a functional feedback loop firstly assess how governance mechanisms evolve and make decisions based on collaborative outputs or contextual changes. Secondly, the criteria assess the capacity of the surveillance system to feed information on the operationalization and impacts of the surveillance back to its leaders and thus the capacity of the latter to adapt.

Second, we worked in depth on evaluation criteria related to information sharing and communication. We introduced the notion of institutional memory, which refers to all information concerning and produced by the multisectoral surveillance system, and we created a criterion to assess its accessibility to surveillance actors and end-users. Evaluation criteria were also added to evaluate the relevance of the information produced by the collaborative surveillance system regarding the collaborative objective(s), as well as the appropriateness of its communication (both in terms of content and means).

Finally, we introduced criteria to address the required sharing of collaboration leadership among stakeholders in a multisectoral surveillance system. We assumed that leadership is evaluated through the existence and operationality of governance mechanisms for the steering, coordination, and technical and scientific support of collaboration. To evaluate if leadership is appropriately distributed across stakeholders, we introduced specific criteria focusing on the representativeness of stakeholders taking part in the governance mechanisms and whether they have an “appropriate voice.” This last term is used to define the active participation of stakeholders in these mechanisms and the fact that there is a simultaneous empowerment of each participant whose respective power is recognized by all (15). There should be a perceived power symmetry with respect to each other (16). In contrast to the term “equal,” the term “appropriate” allows, inside each mechanism, the possibility for some people to have more voice than others and the emergence of champions who catalyze the operationalization of collaboration. All these criteria contribute to the scoring of the functional attribute “shared leadership.”

### The Evaluation Matrix for Collaboration in a Multisectoral Surveillance System

The evaluation matrix is a spreadsheet composed of four sheets.

The first sheet contains the scoring grid for the 75 evaluation criteria (Supplementary Table 4). For each criterion, four possible grades, ranging from 0 to 3, are possible and a detailed definition of the situation according to which each grade should be awarded is provided. The grade, once selected, must be captured in the spreadsheet together with the reasoning and justification that led to the selection of the grade.

The second sheet displays the numerical results of evaluation for each attribute and index. The same formula is used for all of them.

$$\begin{array}{l} \frac{\sum_{i\;=\;0}^{n}x_{i}}{3n} \end{array}$$

*x*_*i*_: grade awarded to a criterion contributing to the definition of the attribute/index.
n: the number of criteria contributing to the definition of the attribute/index and relevant to collaboration under evaluation.
3: the highest score obtained by the criterion when the ideal situation is met.

If some criteria are deemed non-relevant during the evaluation, then they are not included in the scoring of their corresponding attribute or index.

Once the scoring is done, the spreadsheet automatically produces three graphical representations of the evaluation results in the third sheet. Different chart types help to differentiate easily the three levels of evaluation obtained: organization at a microlevel; organization at a macrolevel; and functions. The experience of the OASIS tool using the same principles shows, after more than 25 assessments of surveillance systems, that this option is practically efficient.

**Figure 3:**
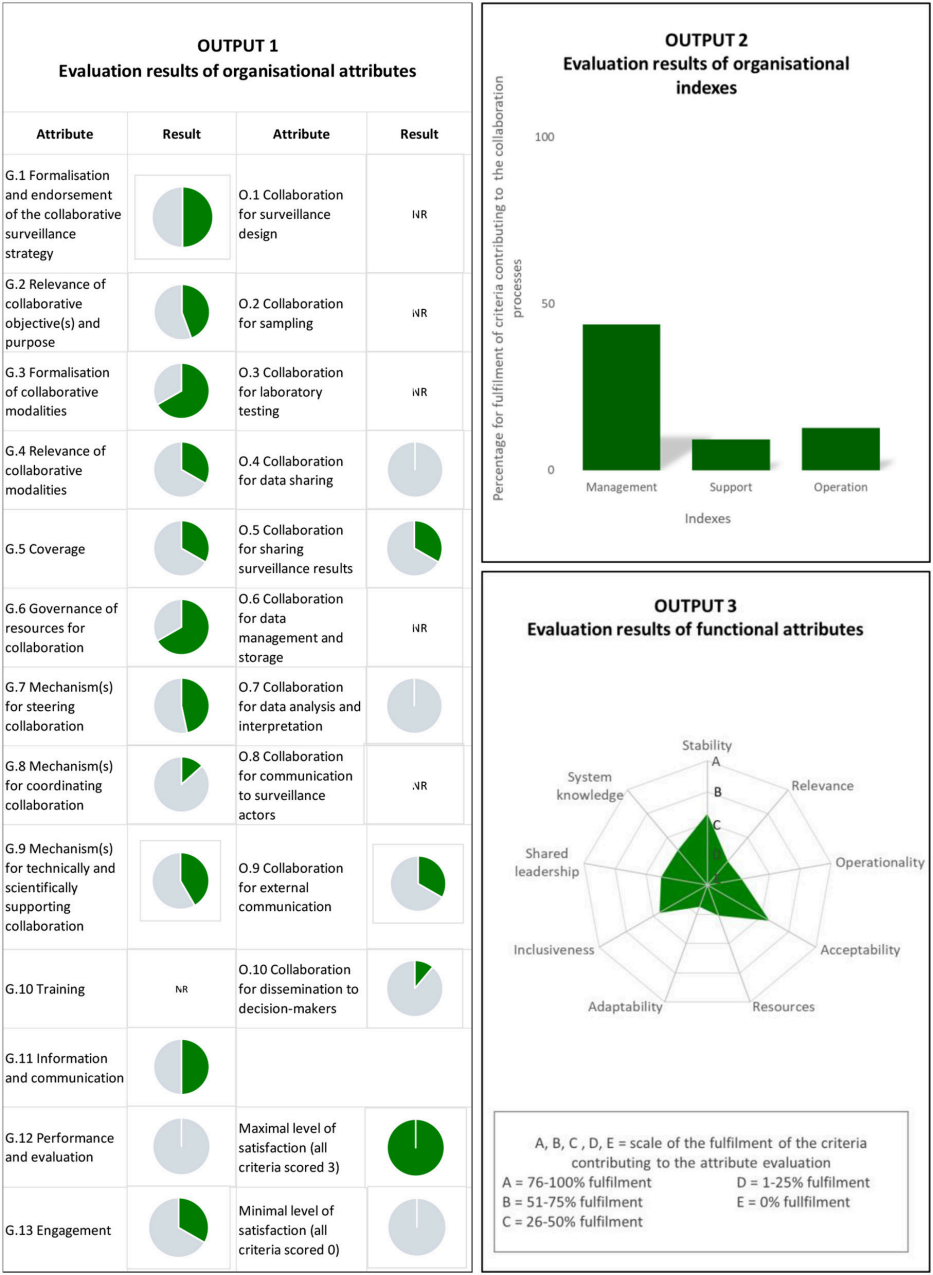
Graphical representations of the evaluation results of the attributes and indexes (examples). NR = the attribute is not relevant to the multi-sectoral surveillance system under evaluation.

The first display represents the evaluation results for the 23 organizational attributes (13 governance and 10 operational attributes). The result for each attribute can be visualized in a pie graph. This graphical format was considered the most appropriate graphical representation to display many individual results (up to 23) and to distinguish easily the attributes evaluated from the ones that were not. Each colored area within a pie chart represents the attribute's level of compliance regarding an “ideal” situation where all evaluation criteria are fully completed. This display provides a visual representation of the level of satisfaction for the organizational attributes, both at the governance and operational levels. It allows to identify easily the weak parts of the collaborative organization (12). The matrix offers an easy means of tracking the criteria that contribute to the scoring of each attribute (sheet 2) to better understand the reasoning behind the scoring and to determine how the different criteria impact the attribute's grade. The second display represents the evaluation results of the indexes. Results of the three indexes are expressed in percentage of compliance of the situation as compared to an “ideal” situation where all criteria score 3. They are displayed in a single histogram. This display illustrates the level of satisfaction regarding the collaborative effort's organization at a macrolevel, from a management, support, and operational point of view. The use of the histogram allows for the visualization of these three highly aggregated evaluation results at a glance and enables an easy comparison of the respective level of satisfaction of the indexes. The last display represents the evaluation results of the nine functional attributes on a spider chart. We considered that an overall spider chart was the most appropriate graphical representation to display results of attributes, which are correlated through common evaluation criteria. Furthermore, it facilitates the analysis of the balance between the different collaborative functions. Results are expressed on a five-tiered scale, from A to E corresponding to the level of satisfaction for each core collaborative function. Grade A corresponds to a level ranging from 76 to 100%, meaning that almost all criteria supporting the evaluation of the attribute scored 3, while grade E corresponds to 0%, meaning that they all scored 0. B, C, and D are intermediate levels of satisfaction, 51–75, 26–50, and 1–25%, respectively. This graphical layout shows the quality of the collaborative effort within the multisectoral surveillance system. It can help to identify the specific collaborative functions that need to be strengthened to make the system more effective. Figure 3 presents the three graphical outputs based on virtual data.

The last sheet contains all the formula to obtain the scoring of attributes and indexes, as well as the graphical representations of the evaluation results.

The matrix is available in the Supplementary Materials.

## Discussion

The proposed matrix is a semiquantitative evaluation matrix developed for an in-depth analysis of the organization, implementation, and functionality of collaboration taking place in a multisectoral surveillance system.

This is a first step in the creation of a fully standalone tool. To this end, guidance must be developed to help evaluators to collect the information needed for scoring criteria, and to guide them through the scoring process and the interpretation of the graphical outputs of evaluation results. Furthermore, the most relevant constitution of the evaluation team and the method used to score evaluation criteria must be determined. Indeed, some criteria might be scored very differently across stakeholders and end-users with various backgrounds, perceptions, and expectations. Nevertheless, the use of the scoring guide is limiting this risk of lack of standardization. The current matrix design is currently very close to that of the OASIS tool, and this proximity to a familiar tool could potentially make the matrix easier to use for evaluators, especially if deployed in synergy with the OASIS tool to globally evaluate the multisectoral surveillance system. To assess its practical application, the tool should be field-tested across different multisectoral surveillance systems targeting various hazards, in different epidemiological and socio-economical contexts, and under various governance and institutional organizations of collaboration.

Although the matrix can be used independently if there is a need to focus on collaboration only, it can be combined with surveillance attributes within existing evaluation tools for an overall assessment of the multisectoral surveillance system.

In its current form, the matrix has three main limitations. First, the evaluation is based on a semiquantitative method to score criteria; this is undoubtedly marked by subjectivity despite the development of the scoring guide. Second, the current matrix does not evaluate the effectiveness of collaboration, nor its impacts and cost, which are crucial parameters for decision-makers (2, 17, 18). Third, the selection of the criteria to support the evaluation of attributes or indexes has not been validated by experts. Furthermore, the scoring method leading to evaluation results of attributes and indexes is assuming that all criteria are of equal importance and may be questioned.

In a multisectoral surveillance system, the collaborative process may occur in several cross-cutting dimensions (sectoral domains, disciplines, professions, decision-making scales), as described by Bordier et al. (13). All these dimensions, when relevant, can be considered and evaluated with the matrix.

The central objective of the matrix is to evaluate collaboration; therefore, some attributes might not be specific to multisectoral surveillance systems, as collaboration can occur in other types of surveillance. For instance, governance attributes related to communication and information sharing are relevant to any surveillance system. However, they are of particular importance in a multisectoral surveillance system due to the multiplicity of stakeholders involved and thus the diversity of background, knowledge, and expectations. Furthermore, issues around the multiplicity of data sources and data ownership are more complicated (19, 20) and need to be addressed properly in all situations.

The evaluation matrix focuses on the quality of collaboration and does not evaluate the performance of the multisectoral surveillance system. However, the evaluation of collaboration cannot be completely disconnected from sectoral surveillance organization and performance, as certain collaborative characteristics are impacted by the settings and capacities in the different domains covered by the multisectoral surveillance system. Therefore, certain collaborative attributes support the evaluation of collaboration within its context. For instance, any evaluation of information generated by collaboration must equally consider the information produced by the sectoral components constituting the multisectoral surveillance system, as it may impact the quality and relevance of the information produced by collaboration regarding the collaborative objective. To evaluate the quality of collaborative activity outputs in terms of its capacity to reach the collaborative objective, the evaluation criteria must also focus on sectoral capacities. Indeed, collaborative activities might be hampered by poor sectoral capacities or by a sectoral organization that is not tailored to support collaborative modalities. For instance, in a multisectoral system where the agreed collaborative modality is that of joint data analysis, the collaborative output might not be achieved because of a lack of comparability across data sets, due to the poor quality of data produced by one sector, or as a result of the inappropriateness of surveillance design in one sectoral component.

The evaluation matrix does not aim to evaluate the degree of integration achieved in a given multisectoral surveillance system. The aim is to qualify the degree of integration that the multisectoral surveillance system seeks to achieve, to assess if this is coherent with the collaborative context, and whether the collaborative modalities and activities designed and implemented are appropriate to achieve it. For instance, in a multisectoral surveillance system, separate institutions may independently supervise surveillance components in their respective jurisdictions and may decide to restrict the collaborative effort to the sharing of information on surveillance results to keep each other updated. Although this modality can be considered as a low level of collaboration, it might be the most relevant to the expected collaborative objective and the epidemiological and socio-economic context. The evaluation matrix will help, without *a priori* consideration, to determine if the collaborative modalities are appropriate and well-operationalized enough to meet the pursued objective in a given context.

However, the aim of any collaborative surveillance system is to integrate different areas of knowledge, competencies, and type of expertise, to improve the effectiveness of the surveillance compared to the effectiveness of several surveillance components operating in silos. Consequently, the matrix enables the evaluation of this integration through the assessment of the existence and relevance of the information produced and of the way it is shared and communicated internally. Furthermore, if some collaborative modalities have been planned for the sharing of surveillance results or data, and/or for the integration of different surveillance data sources for joint analysis, then specific operational attributes can be used to evaluate whether this integration is relevant and appropriate for meeting the collaborative objective. All the criteria related to the quality and integration of information are used to evaluate the functional attribute “System knowledge.” However, at this stage, our evaluation matrix does not allow the evaluation of the impact of this information, as discussed previously.

The expert-opinion elicitation allowed the revision and validation of attributes. The methodology was simple and based on an online questionnaire for the first round, followed by video conference with a selected panel of experts for the second round. Hence, it does not strictly follow established expert-opinion elicitation methodology, which pays specific attention to reducing the bias linked to experts, namely by validating the data they provide before its usage. However, in our study, we considered that the validity of information obtained from experts relied mainly on the diversity of relevant opinions retrieved, which depended on (i) the number of respondents; (ii) their expertise in OH surveillance; and (iii) their representativeness in terms of disciplines, professions, and working organizations. The response rate of the questionnaire was quite low: 39 people answered the questionnaire as compared with the 256 who were contacted in the first instance and asked to disseminate it through their network. This is partly compensated, however, by the fact that 85% of these respondents declared they had strong expertise in the field of OH and surveillance. The major concern is the bias of the study toward respondents' backgrounds and disciplines, as most of them declared they were veterinarians and epidemiologists. The original selection process was supposed to limit this bias by selecting the experts through the articles identified by the systematic literature review (9) and contacting experts working in two major research consortiums focusing on integrated approaches: the NEOH consortium working on the evaluation of OH and the RISKSUR multidisciplinary consortium working on the integrated approach for animal health surveillance evaluation. Two hypotheses may be advanced to explain this observation: (i) currently the field of OH surveillance is predominantly led by veterinarians with epidemiological expertise; or (ii) other professions and disciplines involved in OH surveillance do not publish their work or they use terminology that was not covered by the algorithms used in the systematic literature review (9).

During the two rounds of expert-opinion elicitation, two core collaborative characteristics were considered to be insufficiently addressed in the evaluation matrix, and were extensively discussed by the experts: the political will to establish a multisectoral surveillance system and the existence of champions who can drive the operationalization of collaborative efforts.

As far as we have been able to establish, political will is required when the multisectoral surveillance system is initiated and/or coordinated by competent authorities, to ensure that appropriate support will be provided to institutions in charge of implementing collaborative efforts. However, multisectoral surveillance systems might be established outside any legal framework, within an academic network for instance. As the evaluation matrix is aimed to be generic and applicable to any system, the notion of political will is not appropriate in this context. However, whatever the ownership of surveillance systems, a collaborative policy or strategy must be established to provide a framework for the governance and operation of collaboration. To this end, we introduced an attribute related to the formalization and endorsement of the collaborative surveillance strategy. Through this attribute, the matrix evaluates the political will for collaboration in official multisectoral surveillance systems, where the strategy must be endorsed at a high political level.

We acknowledged that the existence of champions might be necessary in most of the multisectoral surveillance systems to push the operationalization of collaboration ahead. However, we did not consider that a specific attribute was required to evaluate the existence of champions. Indeed, the matrix enables the evaluation of collaborative modality implementation, which might be affected by several factors including the lack of champions to foster the operationalization of the collaborative effort. During the evaluation process, evaluators must assess if this fact hampers the multisectoral surveillance system.

Even among the small community of experts reached through this expert-opinion elicitation, two distinct lines of thought were identified. On one hand, some experts defend the fact that integration is directly proportional to the degree of OH-ness (11) and of cost-effectiveness. They advocate for the supervision of surveillance systems by a single and separate coordinating unit, in charge of all surveillance domains. This governance model is expected to avoid funding inequities that may exist across sectoral jurisdictions by putting resource allocation and planning under the responsibility of a central authority, which would be blind to sectoral mandates in its decision-making. On the other hand, certain respondents emphasized that each sector should be responsible for surveillance in the domains that fall under its respective jurisdiction. Here, the success of OH surveillance relies on the identification of synergies across components that could be brought together more effectively for optimized surveillance. The core element is collaboration and willingness across sectors to identify areas of harmonization and synchronization for surveillance activities. Furthermore, establishing a multisectoral organization in charge of all surveillance components bears the risk that collaboration may take place mainly within this organization, and not between sectors in general.

Our conclusions on the characterization of, and successful collaboration within, multisectoral surveillance systems, which forms the basis of the development of the evaluation matrix, are close to those of other conceptual frameworks that address collaboration.

For instance, D'Amour et al. (21) provide the conceptual basis for inter-professional collaboration in the context of health services. Even though this type of collaboration is not cross-sectoral and aims at delivering better health care services, many analogies exist with multisectoral collaboration for health surveillance. Firstly, the rationale motivating collaboration is quite similar. The increasing complexity of health problems is leading to an increased mutual dependence between different health professionals. This calls for inter-professional collaboration, which aims to improve effectiveness by maximizing individual contributions. The outputs of collaboration are expected to exceed the sum of inputs from each discipline. This is in accordance with the fact that multisectoral surveillance is expected to produce more value than sectoral surveillance components operating independently (2). Secondly, the core concepts used to describe inter-professional collaboration are close to the key features of multisectoral collaboration that we used to develop the attributes in our evaluation matrix: sharing (in terms of responsibilities, decision-making, common philosophy and values, planning, and intervention); partnership (based on open and honest communication and mutual trust and respect); interdependency (underlining the mutual dependence of actors to reach a common goal); power (which needs to be shared across team members); and evolving processes (which describes collaboration as a dynamic and interactive process). Additionally, a review of the literature (22) underlined determinants for successful inter-professional collaboration in health that are similar to the ones we identified for multisectoral collaboration in health surveillance (9, 13): provision of opportunities to support the engagement of individuals in collaborative efforts; allocation of specific financial resources; existence of formalized coordination mechanisms; willingness to collaborate; and trust and mutual respect among individuals.

The analysis of other conceptual frameworks for collaboration revealed some similarities with our findings for multisectoral collaboration in health surveillance (21). The concept of team work underlined that varying degrees of collaboration can happen within a team, ranging from the full autonomy of professionals practicing independently, to a narrow individual autonomy in favor of an autonomous integrated team. Furthermore, formalization is recognized as a crucial element for the implementation of collaboration as it provides an articulated framework for inter-professional work. Finally, operational and functional collaboration is usually associated with strong leadership.

## Conclusion

This evaluation matrix is the first to allow an in-depth analysis of collaboration within a multisectoral surveillance system. As collaboration across sectors and disciplines is increasingly promoted for the development of more efficient surveillance systems, the need has grown to develop capacities to evaluate the quality of this collaboration, and its appropriateness regarding the objective and context. The matrix enables the evaluation of collaboration by assessing satisfaction from different angles, namely: (i) the collaboration's organization at a microlevel with regards to attributes relating to specific collaborative characteristics; (ii) the collaboration's organization at a macrolevel with regards to indexes encompassing a wide range of elements contributing to the same process; and (iii) the collaboration's functions with regards to attributes reflecting core collaborative functionalities. The evaluation attributes have been validated by a group of experts in the field of OH surveillance, and converge with those defined as characterizing collaboration in other activity fields, such as inter-professional collaboration in health care facilities. Indeed, collaboration is not specific to multisectoral surveillance systems, and the attributes developed for the purpose of this matrix could be efficiently used to assess collaboration in other surveillance settings and, after adaptation, even in other multistakeholder systems.

The tool still needs to be finalized through field-testing and the development of a detailed framework to standardize its application. Once these steps are completed, the tool will enable the evaluation of a collaboration's capacity to achieve its goal in a multisectoral setting, as well as its strengths and weaknesses in terms of organization, implementation, and functionality. Evaluation results could then be used to support the development of recommendations to improve the quality and appropriateness of collaboration.

## Author Contributions

MB and CD designed and coordinated the study, analyzed the results, and drafted the manuscript. DN participated in the collection and analysis of data. FG and PH initiated the study, participated in its design and coordination, and contributed to the analysis and interpretation of results, and have equally contributed to the drafting of the manuscript. All authors read and approved the final manuscript.

### Conflict of Interest Statement

The authors declare that the research was conducted in the absence of any commercial or financial relationships that could be construed as a potential conflict of interest.

## Footnotes

^1^http://preparednessandresponse.org/news/mali-measures-progress-first-planning-performance-assessment/

^2^Standard *FD X 50-176: Management tools—Processes management—Guidelines. AFNOR*, August 2017.
