# Supplementary material for: One Health Surveillance: A Matrix to Evaluate Multisectoral Collaboration
Source: Front Vet Sci. 2019 Apr 24;6:109. doi: 10.3389/fvets.2019.00109 (PMC6492491; doi:10.3389/fvets.2019.00109)
Supplement: Supplementary file 1 [file Table_1.docx]

Supplementary table 1. List of organisational attributes of collaboration within a multi-sectoral surveillance system, at the governance (G) and operational (O) levels, as well as the criteria for their evaluation.

| Name of the attribute | Definition of the attribute | Evaluation criteria contributing to the attribute |
| --- | --- | --- |
| G.1 Formalisation and endorsement of the collaborative surveillance strategy | Formalisation of the rationale, objective(s) and purpose of collaboration for the multi-sectoral surveillance system as well as the surveillance actors’ area of actions and endorsement by relevant stakeholders involved from all sectors, disciplines and decision-making scales. | 1. Formalisation of rationale behind the willingness to collaborate for surveillance. |
|  |  | 2. Formalisation of the objective(s) and purpose of collaboration for surveillance. |
|  |  | 3. Formalisation of the surveillance actor’s areas of action in the multi-sectoral surveillance system, i.e. the tasks they are assigned regarding collaboration and coordination of sectoral surveillance. |
|  |  | 4. Endorsement of the documents where the rationale, the objective(s) and purpose of collaboration, and the areas of actions by relevant stakeholders from different sectors, disciplines and decision scales involved. |
| G.2 Relevance of collaborative objective(s) and purpose | Relevance of the collaborative objective(s) and purpose regarding stakeholders' expectations, the epidemiological and socio-economic context, the international/regional guidance (regulations, recommendations, standards). | 5. Relevance of the collaborative objective(s) and purpose regarding actors and end-users' expectations (including meeting the sectoral objectives). |
|  |  | 6. Relevance of the collaborative objective(s) and purpose regarding the epidemiological, socio-political and economic context. |
|  |  | 7. Relevance of the collaborative objective(s) and purpose regarding the international/regional guidance (regulations, recommendations, standards). |
| G.3 Formalisation of collaborative modalities | Description of collaboration in terms of modalities (area and degree of collaboration), and role and responsibilities of surveillance actors. | 8. Formalisation of the collaborative modalities, i.e. the area of collaboration (steps of the surveillance process) and the degree of collaboration |
|  |  | 9. Formalisation of roles and responsibilities of actors involved in collaborative modalities. |
|  |  | 10. Endorsement of the documents -formalising collaborative modalities, and role and responsibilities of surveillance actors involved- by all stakeholders from different sectors, disciplines and decision scales involved OR consistency of documents’ contents across the institutions. |
| G.4 Relevance of collaborative modalities | Relevance of the collaborative modalities (area and degree of collaboration) across the different dimensions regarding collaborative objective(s) and context (including sectoral surveillance capacities) | 11. Relevance of the collaborative modalities regarding the collaborative objective(s) and context (including sectoral surveillance capacities) |
| G.5 Coverage | Relevance of the dimensions and data sources covered by the multi-sectoral surveillance system regarding the collaborative objective(s) and context. | 12. Relevance of the collaborative dimensions (sectors, disciplines, decision making scales, professions) considered in the multi-sectoral surveillance system regarding the collaborative objective(s) and context. |
|  |  | 13. Relevance of the data sources included in the multi-sectoral surveillance system regarding the collaborative objective(s) and context. |
| G.6 Governance of resources for collaboration | Definition of resource allocation mechanisms in the collaborative strategy and allocation of relevant resources for the implementation of collaborative modalities. | 14. Definition of specific mechanisms for financial, material and human resources allocation in the collaborative strategy. |
|  |  | 15. Allocation of relevant financial, material and human resources for the implementation of collaborative modalities. |
|  |  | 16. Adequation between areas of action, and roles and responsibilities assigned in the multi-sectoral surveillance system (collaborative and sectoral activities) regarding professional competencies. |
| G.7 Mechanism(s) for steering collaboration | Existence of appropriate and functional mechanism(s), including feed-back loop, for steering collaboration in the multi-sectoral surveillance system. | 17. Existence and formalisation of mechanism(s) for steering collaboration in the multi-sectoral surveillance system. |
|  |  | 18. Representativeness of all appropriate actors and end-users from relevant sectors, decisions scales and disciplines in the steering mechanism(s) for collaboration (inclusion, participation and appropriate voice). |
|  |  | 19. Operationality of mechanism(s) for steering collaboration including the capacity to advocate for change. |
|  |  | 20. Existence of appropriate feed-back loop in mechanism(s) for steering collaboration. |
|  |  | 21. Availability of all appropriate resources to support mechanism(s) for steering collaboration. |
| G.8 Mechanism(s) for coordinating collaboration | Existence of appropriate and functional mechanism(s), including feed-back loop, for coordinating collaboration in the multi-sectoral surveillance system. | 22. Existence and formalisation of mechanism(s) for coordinating collaboration in the multi-sectoral surveillance system. |
|  |  | 23. Representativeness of all appropriate actors and end-users from relevant sectors, decisions scales and disciplines in the coordinating mechanism(s) for collaboration (inclusion, participation and appropriate voice). |
|  |  | 24. Operationality of mechanism(s) for coordinating collaboration including the capacity to advocate change. |
|  |  | 25. Existence of appropriate feed-back loop in mechanism(s) for coordinating collaboration. |
|  |  | 26. Availability of all appropriate resources to support mechanism(s) for coordinating collaboration. |
| G.9 Mechanism(s) for technically and scientifically supporting collaboration | Existence of appropriate and functional mechanism(s), including feed-back loop, to technically and scientifically support collaboration in the multi-sectoral surveillance system. | 27. Existence and formalisation of mechanism(s) for supporting scientifically and technically collaboration in the multi-sectoral surveillance system. |
|  |  | 28. Representativeness of all appropriate actors from relevant sectors, decisions scales and disciplines for supporting scientifically and technically collaboration (inclusion, participation and appropriate voice). |
|  |  | 29. Operationality of mechanism(s) for supporting scientifically and technically collaboration including the capacity to advocate for change. |
|  |  | 30. Existence of appropriate feedback loop for supporting scientifically and technically collaboration. |
| G.10 Training | Provision of relevant initial and ongoing training for operating actors involved in collaborative activities. | 31. Existence of designed and planned initial training for operating actors involved in collaborative activities. |
|  |  | 32. Accessibility of initial training in relevant timeframe for operating actors involved in collaborative activities. |
|  |  | 33. Relevance of initial training for operating actors involved in collaborative activities with the collaborative modalities and collaborative context. |
|  |  | 34. Existence of designed and planned ongoing training for operating actors involved in collaborative activities. |
|  |  | 35. Accessibility of ongoing training in relevant timeframe for operating actors involved in collaborative activities. |
|  |  | 36. Relevance of ongoing training for operating actors involved in collaborative activities with the collaborative modalities and collaborative context. |
| G.11 Information and communication | Appropriate information production, management and communication. | 37. Existence of an institutional memory including all information related to the rationale of collaboration, to the organisation and functioning of the multi-sectoral surveillance system and to the outputs of the multi-sectoral surveillance system. |
|  |  | 38. Accessibility of the institutional memory to surveillance actors and end-users. |
|  |  | 39. Relevance of the information produced by multi-sectoral surveillance system regarding the collaborative objective(s). |
|  |  | 40. Appropriateness of the communication (both in terms of content and means) of the information produced by the multi-sectoral surveillance system to surveillance actors and end users. |
| G.12 Performance and evaluation | Existence of specific performance indicators of collaboration routinely used and of periodic external evaluations of collaboration | 41. Existence and relevance of specific performance indicators of collaboration routinely used. |
|  |  | 42. Existence of periodic external evaluation of collaboration or of the multi-sectoral surveillance system (including evaluation of collaboration). |
|  |  | 43. Existence of periodic internal evaluation of collaboration or of the multi-sectoral surveillance system (including evaluation of collaboration). |
|  |  | 44. Implementation of corrective measures, if deemed necessary following performance monitoring and evaluation results |
| G.13 Engagement | Engagement of actors in their assigned areas of action, role and responsibilities in the multi-sectoral surveillance system | 45. Engagement of actors in their assigned areas of action, role and responsibilities in the multi-sectoral surveillance system |
| O.1 Collaboration for surveillance design | Implementation of appropriate and functional collaborative activities for the design of the surveillance programme (ex: selection of hazards and population under surveillance, data format, etc.). | 46. Relevance of the collaborative activities for surveillance design regarding the collaborative modalities and context |
|  |  | 47. Appropriateness of the outputs of collaborative activities (including sectoral surveillance capacities) for surveillance design to meet the collaborative objective(s). |
|  |  | 48. Availability of appropriate resources (financial, technical, material and human) to implement the collaborative activities for surveillance design. |
| O.2 Collaboration for sampling | Implementation of appropriate and functional collaborative activities for sampling (ex: joint sample collection campaign, harmonised sample forms). | 49. Relevance of the collaborative activities for sampling regarding the collaborative modalities and context |
|  |  | 50. Appropriateness of the outputs of collaborative activities (including sectoral surveillance capacities) for sampling to meet the collaborative objective(s). |
|  |  | 51. Availability of appropriate resources (financial, technical, material and human) to implement the collaborative activities for sampling. |
| O.3 Collaboration for laboratory testing | Implementation of appropriate and functional collaborative activities for laboratory testing (ex: harmonization of testing methods, interpretation rules, data reporting, etc.). | 52. Relevance of the collaborative activities for laboratory testing regarding the collaborative modalities and context |
|  |  | 53. Appropriateness of the outputs of collaborative activities (including sectoral surveillance capacities) for laboratory testing to meet the collaborative objective(s). |
|  |  | 54. Availability of appropriate resources (financial, technical, material and human) to implement the collaborative activities for laboratory testing. |
| O.4 Collaboration for data sharing | Implementation of appropriate and functional collaborative activities for data sharing (ex: compatibility of information systems across sectors). | 55. Relevance of the collaborative activities for data sharing regarding the collaborative modalities and context |
|  |  | 56. Appropriateness of the outputs of collaborative activities (including sectoral surveillance capacities) for data sharing to meet the collaborative objective(s). |
|  |  | 57. Availability of appropriate resources (financial, technical, material and human) to implement the collaborative activities for data sharing. |
| O.5 Collaboration for sharing surveillance results | Implementation of appropriate collaborative activities for sharing surveillance results (ex: joint inter-sectoral meeting). | 58. Relevance of the collaborative activities for results sharing regarding the collaborative modalities and context |
|  |  | 59. Appropriateness of the outputs of collaborative activities (including sectoral surveillance capacities) for results sharing to meet the collaborative objective(s). |
|  |  | 60. Availability of appropriate resources (financial, technical, material and human) to implement the collaborative activities for results sharing. |
| O.6 Collaboration for data management and storage | Implementation of appropriate and functional collaborative activities for data management/storage (ex: rules for data accessibility, usage and ownership, etc.). | 61. Relevance of the collaborative activities for data management/storage regarding the collaborative modalities and context |
|  |  | 62. Appropriateness of the outputs of collaborative activities (including sectoral surveillance capacities) for data management/storage to meet the collaborative objective(s). |
|  |  | 63. Availability of appropriate resources (financial, technical, material and human) to implement the collaborative activities for data management/storage. |
| O.7 Collaboration for data analysis and interpretation | Implementation of appropriate and functional collaborative activities for data analysis and interpretation (ex: establishment of an inter-sectoral technical working group, etc.). | 64. Relevance of the collaborative activities for data analysis and interpretation regarding the collaborative modalities and context |
|  |  | 65. Appropriateness of the outputs of collaborative activities (including sectoral surveillance capacities) for data analysis and interpretation to meet the collaborative objective(s). |
|  |  | 66. Availability of appropriate resources (financial, technical, material and human) to implement the collaborative activities for data analysis and interpretation. |
| O.8 Collaboration for communication to surveillance actors | Implementation of appropriate collaborative activities for communication of surveillance results to surveillance actors (ex: joint inter-sectoral website, joint inter-sectoral meetings, etc). | 67. Relevance of the collaborative activities for communication of surveillance results to surveillance actors, regarding the collaborative modalities and context |
|  |  | 68. Appropriateness of the outputs of collaborative activities (including sectoral surveillance capacities) for communication of surveillance results to surveillance actors, to meet the collaborative objective(s). |
|  |  | 69. Availability of appropriate resources (financial, technical, material and human) to implement the collaborative activities for communication of surveillance results to surveillance actors,. |
| O.9 Collaboration for external communication | Implementation of appropriate collaborative activities for external communication of surveillance results (ex: joint inter-sectoral website, joint reports, etc). | 70. Relevance of the collaborative activities for external communication of surveillance results regarding the collaborative modalities and context |
|  |  | 71. Appropriateness of the outputs of collaborative activities (including sectoral surveillance capacities) for external communication of surveillance results to meet the collaborative objective(s). |
|  |  | 72. Availability of appropriate resources (financial, technical, material and human) to implement the collaborative activities for external communication of surveillance results. |
| O.10 Collaboration for dissemination to beneficiaries | Implementation of appropriate collaborative activities for dissemination of surveillance results to beneficiaries (ex: joint inter-sectoral reporting to decision-makers, inter-sectoral platform for knowledge transfer, etc.). | 73. Relevance of the collaborative activities for dissemination of surveillance results regarding the collaborative modalities and context. |
|  |  | 74. Appropriateness of the outputs of collaborative activities (including sectoral surveillance capacities) for dissemination of surveillance results to meet the collaborative objective(s). |
|  |  | 75. Availability of appropriate resources (financial, technical, material and human) to implement the collaborative activities for dissemination of surveillance results. |
