# Supplementary material for: One Health Surveillance: A Matrix to Evaluate Multisectoral Collaboration
Source: Front Vet Sci. 2019 Apr 24;6:109. doi: 10.3389/fvets.2019.00109 (PMC6492491; doi:10.3389/fvets.2019.00109)
Supplement: Supplementary file 2 [file Table_2.docx]

Supplementary table 2. List of functional attributes of collaboration within a multi-sectoral surveillance system, at the governance (G) et operational (O) levels, as well as the criteria for their evaluation.

| Name of the attribute | Definition of the attribute | Criteria for the evaluation of the attribute |
| --- | --- | --- |
| Stability | Collaboration is stable in time, it is formalised and endorsed by all relevant stakeholders (surveillance actors and end-users). | 1. Formalisation of rationale behind the willingness to collaborate for surveillance. |
|  |  | 2. Formalisation of the objective(s) and purpose of collaboration for surveillance. |
|  |  | 3. Formalisation of the surveillance actor’s areas of action in the multi-sectoral surveillance system, i.e. the tasks they are assigned regarding collaboration and coordination of sectoral surveillance. |
|  |  | 4. Endorsement of the documents where the rationale, the objective(s) and purpose of collaboration, and the areas of actions by relevant stakeholders from different sectors, disciplines and decision scales involved. |
|  |  | 8. Formalisation of the collaborative modalities, i.e. the area of collaboration (steps of the surveillance process) and the degree of collaboration |
|  |  | 9. Formalisation of roles and responsibilities of actors involved in collaborative modalities. |
|  |  | 10. Endorsement of the documents -formalising collaborative modalities, and role and responsibilities of surveillance actors involved- by all stakeholders from different sectors, disciplines and decision scales involved OR consistency of documents’ contents across the institutions. |
|  |  | 14. Definition of specific mechanisms for financial, material and human resources allocation in the collaborative strategy. |
|  |  | 17. Existence and formalisation of mechanism(s) for steering collaboration in the multi-sectoral surveillance system. |
|  |  | 22. Existence and formalisation of mechanism(s) for coordinating collaboration in the multi-sectoral surveillance system. |
|  |  | 27. Existence and formalisation of mechanism(s) for supporting scientifically and technically collaboration in the multi-sectoral surveillance system. |
|  |  | 31. Existence of designed and planned initial training for operating actors involved in collaborative activities. |
|  |  | 34. Existence of designed and planned ongoing training for operating actors involved in collaborative activities. |
| Relevance | Collaborative strategy, modalities and activities are relevant regarding the collaborative objective and context. | 5. Relevance of the collaborative objective(s) and purpose regarding actors and end-users' expectations (including meeting the sectoral objectives). |
|  |  | 6. Relevance of the collaborative objective(s) and purpose regarding the epidemiological, socio-political and economic context. |
|  |  | 7. Relevance of the collaborative objective(s) and purpose regarding the international/regional guidance (regulations, recommendations, standards). |
|  |  | 11. Relevance of the collaborative modalities regarding the collaborative objective(s) and context (including sectoral surveillance capacities) |
|  |  | 12. Relevance of the dimensions (sectors, disciplines, decision making scales, professions) regarding the collaborative objective(s) and context |
|  |  | 13. Relevance of the data sources regarding the collaborative objective(s) and context. |
|  |  | 46. Relevance of the collaborative activities for surveillance design regarding the collaborative modalities and context |
|  |  | 49. Relevance of the collaborative activities for sampling regarding the collaborative modalities and context |
|  |  | 52. Relevance of the collaborative activities for laboratory testing regarding the collaborative modalities and context |
|  |  | 55. Relevance of the collaborative activities for data sharing regarding the collaborative modalities and context |
|  |  | 58. Relevance of the collaborative activities for results sharing regarding the collaborative modalities and context |
|  |  | 61. Relevance of the collaborative activities for data management/storage regarding the collaborative modalities and context |
|  |  | 64. Relevance of the collaborative activities for data analysis and interpretation regarding the collaborative modalities and context |
|  |  | 67. Relevance of the collaborative activities for communication of surveillance results to surveillance actors, regarding the collaborative modalities and context |
|  |  | 70. Relevance of the collaborative activities for external communication of surveillance results regarding the collaborative modalities and context |
|  |  | 73. Relevance of the collaborative activities for dissemination of surveillance results regarding the collaborative modalities and context |
| Operationality | The governance of collaboration is operational, and collaboration is effectively implemented to meet the surveillance objective. | 19. Operationality of mechanism(s) for steering collaboration including the capacity to advocate for change. |
|  |  | 20. Existence of appropriate feed-back loop in mechanism(s) for steering collaboration. |
|  |  | 24. Operationality of mechanism(s) for coordinating collaboration including the capacity to advocate change. |
|  |  | 25. Existence of appropriate feed-back loop in mechanism(s) for steering collaboration. |
|  |  | 29. Operationality of mechanism(s) for supporting scientifically and technically collaboration including the capacity to advocate for change. |
|  |  | 30. Existence of appropriate feedback loop for supporting scientifically and technically collaboration. |
|  |  | 39. Relevance of the information produced by multi-sectoral surveillance system regarding the collaborative objective(s). |
|  |  | 47. Appropriateness of the outputs of collaborative activities (including sectoral surveillance capacities) for surveillance design to meet the collaborative objective(s). |
|  |  | 50. Appropriateness of the outputs of collaborative activities (including sectoral surveillance capacities) for sampling to meet the collaborative objective(s). |
|  |  | 53. Appropriateness of the outputs of collaborative activities (including sectoral surveillance capacities) for laboratory testing to meet the collaborative objective(s). |
|  |  | 56. Appropriateness of the outputs of collaborative activities (including sectoral surveillance capacities) for data sharing to meet the collaborative objective(s). |
|  |  | 59. Appropriateness of the outputs of collaborative activities (including sectoral surveillance capacities) for results sharing to meet the collaborative objective(s). |
|  |  | 62. Appropriateness of the outputs of collaborative activities (including sectoral surveillance capacities) for data management/storage to meet the collaborative objective(s). |
|  |  | 65. Appropriateness of the outputs of collaborative activities (including sectoral surveillance capacities) for data analysis and interpretation to meet the collaborative objective(s). |
|  |  | 68. Appropriateness of the outputs of collaborative activities (including sectoral surveillance capacities) for communication of surveillance results to surveillance actors, to meet the collaborative objective(s). |
|  |  | 71. Appropriateness of the outputs of collaborative activities (including sectoral surveillance capacities) for external communication of surveillance results to meet the collaborative objective(s). |
|  |  | 74. Appropriateness of the outputs of collaborative activities (including sectoral surveillance capacities) for dissemination of surveillance results to meet the collaborative objective(s). |
| Acceptability | Surveillance actors demonstrate trust into the system, mutual understanding and willingness to collaborate. The objective(s) of collaboration and outputs of the multi-sectoral surveillance system meet stakeholders (surveillance actors and end-users) expectations. | 4. Endorsement of the documents where the rationale, the objective(s) and purpose of collaboration, and the areas of actions by relevant stakeholders from different sectors, disciplines and decision scales involved. |
|  |  | 5. Relevance of the collaborative objective(s) and purpose regarding actors and end-users' expectations (including meeting the sectoral objectives). |
|  |  | 10. Endorsement of the documents -formalising collaborative modalities, and role and responsibilities of surveillance actors involved- by all stakeholders from different sectors, disciplines and decision scales involved OR consistency of documents’ contents across the institutions. |
|  |  | 16. Adequation between areas of action, and roles and responsibilities assigned in the multi-sectoral surveillance system (collaborative and sectoral activities) regarding professional competencies. |
|  |  | 32. Accessibility of initial training in relevant timeframe for operating actors involved in collaborative activities. |
|  |  | 33. Relevance of initial training for operating actors involved in collaborative activities with the collaborative modalities and context. |
|  |  | 35. Accessibility of ongoing training in relevant timeframe for operating actors involved in collaborative activities. |
|  |  | 36. Relevance of ongoing training for operating actors involved in collaborative activities with the collaborative modalities and context. |
|  |  | 38. Accessibility of the institutional memory to surveillance actors and end-users. |
|  |  | 40. Appropriateness of the communication (both in terms of content and means) of the information produced by the multi-sectoral surveillance system to surveillance actors and end users. |
|  |  | 45. Engagement of actors in their assigned areas of action, role and responsibilities in the multi-sectoral surveillance system |
| Resources | The mechanisms for resources allocation are defined. The resources are appropriate and available for the effective implementation of activities of collaboration. | 14. Definition of specific mechanisms for financial, material and human resources allocation in the collaborative strategy. |
|  |  | 15. Allocation of relevant financial, material and human resources for the implementation of collaborative modalities. |
|  |  | 21. Availability of all appropriate resources to support mechanism(s) for steering collaboration. |
|  |  | 26. Availability of all appropriate resources to support mechanism(s) for coordinating collaboration. |
|  |  | 32. Accessibility of initial training in relevant timeframe for operating actors involved in collaborative activities. |
|  |  | 35. Accessibility of ongoing training in relevant timeframe for operating actors involved in collaborative activities. |
|  |  | 48. Availability of appropriate resources (financial, technical, material and human) to implement the collaborative activities for surveillance design. |
|  |  | 51. Availability of appropriate resources (financial, technical, material and human) to implement the collaborative activities for sampling. |
|  |  | 54. Availability of appropriate resources (financial, technical, material and human) to implement the collaborative activities for laboratory testing. |
|  |  | 57. Availability of appropriate resources (financial, technical, material and human) to implement the collaborative activities for data sharing. |
|  |  | 60. Availability of appropriate resources (financial, technical, material and human) to implement the collaborative activities for results sharing. |
|  |  | 63. Availability of appropriate resources (financial, technical, material and human) to implement the collaborative activities for data management/storage. |
|  |  | 66. Availability of appropriate resources (financial, technical, material and human) to implement the collaborative activities for data analysis and interpretation. |
|  |  | 69. Availability of appropriate resources (financial, technical, material and human) to implement the collaborative activities for communication of surveillance results to surveillance actors,. |
|  |  | 72. Availability of appropriate resources (financial, technical, material and human) to implement the collaborative activities for external communication of surveillance results. |
|  |  | 75. Availability of appropriate resources (financial, technical, material and human) to implement the collaborative activities for dissemination of surveillance results. |
| Adaptability | Collaboration can adapt and evolve upon changes in governance modalities, knowledge and context. | 19. Operationality of mechanism(s) for steering collaboration including the capacity to advocate for change. |
|  |  | 20. Existence of appropriate feed-back loop in mechanism(s) for steering collaboration. |
|  |  | 24. Operationality of mechanism(s) for coordinating collaboration including the capacity to advocate change. |
|  |  | 25. Existence of appropriate feed-back loop in mechanism(s) for coordinating collaboration. |
|  |  | 29. Operationality of mechanism(s) for supporting scientifically and technically collaboration including the capacity to advocate for change. |
|  |  | 30. Existence of appropriate feedback loop for supporting scientifically and technically collaboration. |
|  |  | 41. Existence and relevance of specific performance indicators of collaboration routinely used. |
|  |  | 42. Existence of periodic external evaluation of collaboration or of the multi-sectoral surveillance system (including evaluation of collaboration). |
|  |  | 43. Existence of periodic internal evaluation of collaboration or of the multi-sectoral surveillance system (including evaluation of collaboration). |
|  |  | 44. Implementation of corrective measures, if deemed necessary following performance monitoring and evaluation results |
| Inclusiveness | Relevant surveillance actors and end-users participate in governance mechanisms. Roles in collaboration are adequately allocated to actors with regard their mandates and competencies. At the relevant dimensions, corresponding actors and data sources are considered to meet the collaborative objective(s). | 12. Relevance of the collaborative dimensions (sectors, disciplines, decision making scales, professions) considered in the multi-sectoral surveillance system regarding the collaborative objective(s) and context. |
|  |  | 13. Relevance of the data sources included in the multi-sectoral surveillance system regarding the collaborative objective(s) and context. |
|  |  | 16. Adequation between areas of action, and roles and responsibilities assigned in the multi-sectoral surveillance system (collaborative and sectoral activities) regarding professional competencies. |
|  |  | 18. Representativeness of all appropriate actors and end-users from relevant sectors, decisions scales and disciplines in the steering mechanism(s) for collaboration (inclusion, participation and appropriate voice). |
|  |  | 23. Representativeness of all appropriate actors and end-users from relevant sectors, decisions scales and disciplines in the coordinating mechanism(s) for collaboration (inclusion, participation and appropriate voice). |
|  |  | 28. Representativeness of all appropriate actors from relevant sectors, decisions scales and disciplines for supporting scientifically and technically collaboration (inclusion, participation and appropriate voice). |
| Shared leadership | Governance mechanisms are appropriate to guide the operation of collaboration in the multi-sectoral surveillance system. They provide a trustworthy environment where stakeholders can freely express their views and be heard, creating mutual understanding. | 4. Endorsement of the documents where the rationale, the objective(s) and purpose of collaboration, and the areas of actions by all stakeholders from different sectors, disciplines and decision scales involved. |
|  |  | 5. Relevance of the collaborative objective(s) and purpose regarding actors and end-users' expectations (including meeting the sectoral objectives). |
|  |  | 18. Representativeness of all appropriate actors and end-users from relevant sectors, decisions scales and disciplines in the steering mechanism(s) for collaboration (inclusion, participation and appropriate voice). |
|  |  | 19. Operationality of mechanism(s) for steering collaboration including the capacity to advocate for change. |
|  |  | 23. Representativeness of all appropriate actors and end-users from relevant sectors, decisions scales and disciplines in the coordinating mechanism(s) for collaboration (inclusion, participation and appropriate voice). |
|  |  | 24. Operationality of mechanism(s) for coordinating collaboration including the capacity to advocate change. |
|  |  | 28. Representativeness of all appropriate actors from relevant sectors, decisions scales and disciplines for supporting scientifically and technically collaboration (inclusion, participation and appropriate voice). |
|  |  | 29. Operationality of mechanism(s) for supporting scientifically and technically collaboration including the capacity to advocate for change. |
| System knowledge | The multi-sectoral surveillance system has a comprehensive and accessible institutional memory and demonstrates an effective communication system. Stakeholders (surveillance actors and end-users) have access to relevant information about the collaborative surveillance organisation and outputs. Surveillance data and results are shared at a relevant level, with regard to collaborative objective and context. | 12. Relevance of the collaborative dimensions (sectors, disciplines, decision making scales, professions) considered in the multi-sectoral surveillance system regarding the collaborative objective(s) and context. |
|  |  | 13. Relevance of the data sources included in the multi-sectoral surveillance system regarding the collaborative objective(s) and context. |
|  |  | 29. Operationality of mechanism(s) for supporting scientifically and technically collaboration including the capacity to advocate for change. |
|  |  | 33. Relevance of initial training for operating actors involved in collaborative activities with the collaborative modalities and collaborative context. |
|  |  | 36. Relevance of ongoing training for operating actors involved in collaborative activities with the collaborative modalities and collaborative context. |
|  |  | 37. Existence of an institutional memory including all information related to the rationale of collaboration, to the organisation and functioning of the multi-sectoral surveillance system and to the outputs of the multi-sectoral surveillance system. |
|  |  | 38. Accessibility of the institutional memory to surveillance actors and end-users. |
|  |  | 39. Relevance of the information produced by multi-sectoral surveillance system regarding the collaborative objective(s). |
|  |  | 40. Appropriateness of the communication (both in terms of content and means) of the information produced by the multi-sectoral surveillance system to surveillance actors and end users. |
|  |  | 56. Appropriateness of the outputs of collaborative activities (including sectoral surveillance capacities) for data sharing to meet the collaborative objective(s). |
|  |  | 59. Appropriateness of the outputs of collaborative activities (including sectoral surveillance capacities) for results sharing to meet the collaborative objective(s). |
|  |  | 65. Appropriateness of the outputs of collaborative activities (including sectoral surveillance capacities) for data analysis and interpretation to meet the collaborative objective(s). |
