# Supplementary material for: One Health Surveillance: A Matrix to Evaluate Multisectoral Collaboration
Source: Front Vet Sci. 2019 Apr 24;6:109. doi: 10.3389/fvets.2019.00109 (PMC6492491; doi:10.3389/fvets.2019.00109)
Supplement: Supplementary file 3 [file Table_3.docx]

Supplementary table 3. List of indexes for the organisation and operation of collaboration within a multi-sectoral surveillance system, at the governance (G) et operational (O) levels, as well as the criteria for their evaluation.

| Name of the index | Definition of the index | Criteria for the evaluation of the index |
| --- | --- | --- |
| Management index | All elements contributing to the management of collaboration: existence and formalisation of a collaborative strategy, governance mechanisms for steering and coordination, performance monitoring and evaluation. | 01. Formalisation of rationale behind the willingness to collaborate for surveillance. |
|  |  | 02. Formalisation of the objective(s) and purpose of collaboration for surveillance. |
|  |  | 3. Formalisation of the surveillance actor’s areas of action in the multi-sectoral surveillance system, i.e. the tasks they are assigned regarding collaboration and coordination of sectoral surveillance. |
|  |  | 4. Endorsement of the documents where the rationale, the objective(s) and purpose of collaboration, and the areas of actions by relevant stakeholders from different sectors, disciplines and decision scales involved. |
|  |  | 05. Relevance of the collaborative objective(s) and purpose regarding actors and end-users' expectations (including meeting the sectoral objectives). |
|  |  | 06. Relevance of the collaborative objective(s) and purpose regarding the epidemiological, socio-political and economic context. |
|  |  | 07. Relevance of the collaborative objective(s) and purpose regarding the international/regional guidance (regulations, recommendations, standards). |
|  |  | 08. Formalisation of the collaborative modalities, i.e. the area of collaboration (steps of the surveillance process) and the degree of collaboration |
|  |  | 09. Formalisation of roles and responsibilities of actors involved in collaborative modalities. |
|  |  | 10. Endorsement of the documents -formalising collaborative modalities, and role and responsibilities of surveillance actors involved- by all stakeholders from different sectors, disciplines and decision scales involved OR consistency of documents’ contents across the institutions. |
|  |  | 11. Relevance of the collaborative modalities regarding the collaborative objective(s) and context (including sectoral surveillance capacities) |
|  |  | 12. Relevance of the collaborative dimensions (sectors, disciplines, decision making scales, professions) considered in the multi-sectoral surveillance system regarding the collaborative objective(s) and context. |
|  |  | 13. Relevance of the data sources included in the multi-sectoral surveillance system regarding the collaborative objective(s) and context. |
|  |  | 14. Definition of specific mechanisms for financial, material and human resources allocation in the collaborative strategy. |
|  |  | 17. Existence and formalisation of mechanism(s) for steering collaboration in the multi-sectoral surveillance system. |
|  |  | 18. Representativeness of all appropriate actors and end-users from relevant sectors, decisions scales and disciplines in the steering mechanism(s) for collaboration (inclusion, participation and appropriate voice). |
|  |  | 19. Operationality of mechanism(s) for steering collaboration including the capacity to advocate for change. |
|  |  | 20. Existence of appropriate feed-back loop in mechanism(s) for steering collaboration. |
|  |  | 21. Availability of all appropriate resources to support mechanism(s) for steering collaboration. |
|  |  | 22. Existence and formalisation of mechanism(s) for coordinating collaboration in the multi-sectoral surveillance system. |
|  |  | 23. Representativeness of all appropriate actors and end-users from relevant sectors, decisions scales and disciplines in the coordinating mechanism(s) for collaboration (inclusion, participation and appropriate voice). |
|  |  | 24. Operationality of mechanism(s) for coordinating collaboration including the capacity to advocate change. |
|  |  | 25. Existence of appropriate feed-back loop in mechanism(s) for coordinating collaboration. |
|  |  | 26. Availability of all appropriate resources to support mechanism(s) for coordinating collaboration. |
|  |  | 41. Existence and relevance of specific performance indicators of collaboration routinely used. |
|  |  | 42. Existence of periodic external evaluation of collaboration or of the multi-sectoral surveillance system (including evaluation of collaboration). |
|  |  | 43. Existence of periodic internal evaluation of collaboration or of the multi-sectoral surveillance system (including evaluation of collaboration). |
|  |  | 44. Implementation of corrective measures, if deemed necessary following performance monitoring and evaluation results |
|  |  | 45. Engagement of actors in their assigned areas of action, role and responsibilities in the multi-sectoral surveillance system |
| Support index | All elements in place that ensure the smooth operation of collaboration: resources allocation, training, information and communication, technical and scientific support | 15. Allocation of relevant financial, material and human resources for the implementation of collaborative modalities. |
|  |  | 16. Adequation between areas of action, and roles and responsibilities assigned in the multi-sectoral surveillance system (collaborative and sectoral activities) regarding professional competencies. |
|  |  | 27. Existence and formalisation of mechanism(s) for supporting scientifically and technically collaboration in the multi-sectoral surveillance system. |
|  |  | 28. Representativeness of all appropriate actors from relevant sectors, decisions scales and disciplines for supporting scientifically and technically collaboration (inclusion, participation and appropriate voice). |
|  |  | 29. Operationality of mechanism(s) for supporting scientifically and technically collaboration including the capacity to advocate for change. |
|  |  | 30. Existence of appropriate feedback loop for supporting scientifically and technically collaboration. |
|  |  | 31. Existence of designed and planned initial training for operating actors involved in collaborative activities. |
|  |  | 32. Accessibility of initial training in relevant timeframe for operating actors involved in collaborative activities. |
|  |  | 33. Relevance of initial training for operating actors involved in collaborative activities with the collaborative modalities and collaborative context. |
|  |  | 34. Existence of designed and planned ongoing training for operating actors involved in collaborative activities. |
|  |  | 35. Accessibility of ongoing training in relevant timeframe for operating actors involved in collaborative activities. |
|  |  | 36. Relevance of ongoing training for operating actors involved in collaborative activities with the collaborative modalities and collaborative context. |
|  |  | 37. Existence of an institutional memory including all information related to the rationale of collaboration, to the organisation and functioning of the multi-sectoral surveillance system and to the outputs of the multi-sectoral surveillance system. |
|  |  | 38. Accessibility of the institutional memory to surveillance actors and end-users. |
|  |  | 40. Appropriateness of the communication (both in terms of content and means) of the information produced by the multi-sectoral surveillance system to surveillance actors and end users. |
|  |  | 48. Availability of appropriate resources (financial, technical, material and human) to implement the collaborative activities for surveillance design. |
|  |  | 51. Availability of appropriate resources (financial, technical, material and human) to implement the collaborative activities for sampling. |
|  |  | 54. Availability of appropriate resources (financial, technical, material and human) to implement the collaborative activities for laboratory testing. |
|  |  | 57. Availability of appropriate resources (financial, technical, material and human) to implement the collaborative activities for data sharing. |
|  |  | 60. Availability of appropriate resources (financial, technical, material and human) to implement the collaborative activities for results sharing. |
|  |  | 63. Availability of appropriate resources (financial, technical, material and human) to implement the collaborative activities for data management/storage. |
|  |  | 66. Availability of appropriate resources (financial, technical, material and human) to implement the collaborative activities for data analysis and interpretation. |
|  |  | 69. Availability of appropriate resources (financial, technical, material and human) to implement the collaborative activities for communication of surveillance results to surveillance actors. |
|  |  | 72. Availability of appropriate resources (financial, technical, material and human) to implement the collaborative activities for external communication of surveillance results. |
|  |  | 75. Availability of appropriate resources (financial, technical, material and human) to implement the collaborative activities for dissemination of surveillance results. |
| Operation index | All collaborative activities for surveillance (from surveillance design to results dissemination) that generate the relevant collaborative surveillance outputs to meet the collaborative objective(s) and purpose(s) | 39. Relevance of the information produced by multi-sectoral surveillance system regarding the collaborative objective(s). |
|  |  | 46. Relevance of the collaborative activities for surveillance design regarding the collaborative modalities and context |
|  |  | 47. Appropriateness of the outputs of collaborative activities (including sectoral surveillance capacities) for surveillance design to meet the collaborative objective(s). |
|  |  | 49. Relevance of the collaborative activities for sampling regarding the collaborative modalities and context |
|  |  | 50. Appropriateness of the outputs of collaborative activities (including sectoral surveillance capacities) for sampling to meet the collaborative objective(s). |
|  |  | 52. Relevance of the collaborative activities for laboratory testing regarding the collaborative modalities and context |
|  |  | 53. Appropriateness of the outputs of collaborative activities (including sectoral surveillance capacities) for laboratory testing to meet the collaborative objective(s). |
|  |  | 55. Relevance of the collaborative activities for data sharing regarding the collaborative modalities and context |
|  |  | 56. Appropriateness of the outputs of collaborative activities (including sectoral surveillance capacities) for data sharing to meet the collaborative objective(s). |
|  |  | 58. Relevance of the collaborative activities for results sharing regarding the collaborative modalities and context |
|  |  | 59. Appropriateness of the outputs of collaborative activities (including sectoral surveillance capacities) for results sharing to meet the collaborative objective(s). |
|  |  | 61. Relevance of the collaborative activities for data management/storage regarding the collaborative modalities and context |
|  |  | 62. Appropriateness of the outputs of collaborative activities (including sectoral surveillance capacities) for data management/storage to meet the collaborative objective(s). |
|  |  | 64. Relevance of the collaborative activities for data analysis and interpretation regarding the collaborative modalities and context |
|  |  | 65. Appropriateness of the outputs of collaborative activities (including sectoral surveillance capacities) for data analysis and interpretation to meet the collaborative objective(s). |
|  |  | 67. Relevance of the collaborative activities for communication of surveillance results to surveillance actors, regarding the collaborative modalities and context |
|  |  | 68. Appropriateness of the outputs of collaborative activities (including sectoral surveillance capacities) for communication of surveillance results to surveillance actors, to meet the collaborative objective(s). |
